# Microbiological Quality and Antimicrobial Resistance of Commercial Probiotic Products for Food-Producing Animals

**DOI:** 10.3390/antibiotics13020148

**Published:** 2024-02-01

**Authors:** Hoang My Tran, Rangsiya Prathan, Si Thu Hein, Rungtip Chuanchuen

**Affiliations:** 1 The International Graduate Course of Veterinary Science and Technology (VST), Faculty of Veterinary Science, Chulalongkorn University, Bangkok 10330, Thailand; hoangmy.bsty@gmail.com; 2 Research Unit in Microbial Food Safety and Antimicrobial Resistance, Department of Veterinary Public Health, Faculty of Veterinary Science, Chulalongkorn University, Bangkok 10330, Thailand; rochun.30@gmail.com (R.P.); sithuhein5343@gmail.com (S.T.H.); 3 Center for Antimicrobial Resistance Monitoring in Foodborne Pathogens (in Cooperation with WHO), Faculty of Veterinary Science, Chulalongkorn University, Bangkok 10330, Thailand

**Keywords:** antimicrobial resistance, food animals, probiotics

## Abstract

Probiotics have been popularly used in livestock production as an alternative to antibiotics. This study aimed to investigate the microbiological quality and phenotypic and genotypic antimicrobial resistance of bacteria in probiotic products sold for food animals. A total of 45 probiotic products were examined for the number of viable cells, species, and antimicrobial susceptibility; the contamination of *Escherichia coli* and *Salmonella*; and the presence of 112 genes encoding resistance to clinically important antimicrobials and transferability of AMR determinants. The results showed that 29 of 45 products (64.4%) were incorrectly labeled in either number of viable cells or bacterial species. None of the tested products were contaminated with *E. coli* and *Salmonella*. A total of 33 out of 64 bacterial isolates (51.6%) exhibited resistance to at least one antimicrobial agent. Of the 45 products tested, 16 (35.5%) carried AMR genes. Almost all AMR genes detected in probiotic products were not correlated to the AMR phenotype of probiotic strains formulated in the products. Three streptomycin-resistant *Lactobacillus* isolates could horizontally transfer their AMR determinants. The findings demonstrated that the probiotic products could serve as reservoirs for the spread of AMR genes and may not yield benefits to animals as claimed. The need for the adequate quality control of probiotic products is highlighted.

## 1. Introduction

In recent decades, the rise in antimicrobial resistance (AMR) in bacteria is one of the significant global public health challenges. The AMR problem impacts upon human, animal, and environmental health and is referred to as a critical One Health issue. Currently, the AMR issue has generated implications in food safety and food security. In food-producing animals, antimicrobials have been widely used for the treatment and prevention of infections and growth promotion [1]. However, the over- and improper use of antimicrobials have resulted in developing resistance in bacteria. Such resistant bacteria may subsequently enter the food chain or transfer their AMR determinants to other bacterial pathogens. A reduction in antimicrobial use in food animals is a milestone in the strategic plan to minimize the emergence and spread of AMR. The use of antibiotic growth promoters in food animal production has been banned in many countries, e.g., EU countries, Canada, the US, China, Thailand, etc., [2] and the use of alternatives to antibiotics to promote health and reduce diseases has gained tremendous interest. Among the antibiotic alternatives, probiotics have been widely used in food animals for a long time. Probiotics are defined as “live microorganisms that, when administered in adequate amounts, confer a health benefit on the host” [3]. Probiotic products commonly contain one or more species belonging to the genera of *Lactobacillus*, *Bacillus*, *Enterococcus*, and *Clostridium*. Although probiotics provide beneficial effects on the health of both livestock and humans, particular concerns associated with their quality and safety have been raised.

Recently, several studies have demonstrated that the actual quality of several commercial probiotic products deviated from the declared label [4]. The common deviations included low levels of viable bacterial cells and misidentified species of microorganisms. The beneficial health effects of probiotics are associated with the number of viable bacterial cells, while their safety are species- and strain-dependent. Therefore, any deviations from the label claims could result in a loss of probiotic quality and benefits. Additionally, we should be aware of the risk of contamination of pathogenic bacteria (e.g., *Salmonella* spp. and *Escherichia coli*); it cannot be underestimated. Therefore, particular concerns have been raised for both the beneficial effects and potential health risks of probiotic products.

The presence of AMR bacteria and determinants in several probiotic products was previously reported [4]. Recent studies demonstrated that probiotic bacteria were resistant to various clinically important antibiotics and carried resistance determinants potentially transferred to commensal microbiota and pathogenic bacteria in the gut through horizontal gene transfer [5,6]. Therefore, the use of such probiotics in animal feed can pose a double-edged sword, leading to a wide distribution of AMR and failure in the implementations for combating AMR.

The regulations of probiotic products for humans and animals differ between countries. In Europe, the Regulation EC No. 1831/2003 was issued for the legislation of the authorization, use, monitoring, labelling, and packaging of feed additives [7]. In addition, the Qualified Presumption as Safe (QPS) was developed by the European Food Safety Authority (EFSA) for the safety assessment of micro-organisms intentionally introduced into human food and animal feed, considering the identity, history of use, virulence, and transferable resistance determinants [8]. In Thailand, the Animal Feed Quality Control Act B.E. 2558 has been launched; however, there is no specific regulation of probiotic products for animals in terms of viable bacterial number, species, and AMR determinants [9]. Most studies on probiotic products for food-producing animals have focused on testing the effectiveness, but not the safety, of probiotics. This will open up the chance of dispersing poor-quality products and introducing AMR determinants into the farms. Therefore, research studies to examine the microbiology quality of, and AMR in, probiotic products that are commercially available for food animals are required.

The aims of this study were to evaluate the microbiological quality and safety of probiotic products used for food animals, including number and species of probiotic bacteria, contamination of pathogenic bacteria, and the presence and transfer of AMR determinants.

## 2. Results

### 2.1. Numbers and Species of Probiotic Bacteria

Overall, 29 products (64.4%) had at least one discrepancy on the label, including lower number of viable cells (*n* = 11), misnaming of bacterial species (*n* = 26), and vague labelling (*n* = 3). The comparison between information given on labels and the observation in this study is shown in Table 1.

Among the 41 products tested number of viable cells, 11 products (26.8%) had a lower number of viable cells than their label claims. No viable *Lactobacillus* was found in some products (P12, P31, and P32). Thirty products (73.2%) contained viable cells approximately equivalent to or exceeded the declared contents, especially the products formulated by *Bacillus* and/or *Enterococcus*.

Out of the 45 products’ identified strains and species, 26 products (57.8%) comprised other species rather than those claimed on the contents. Twenty-two products claimed to contain members of the *B. subtilis* cluster (*B. pumilus*, *B. amyloliquefaciens*, and *B. atrophaeus*). Products P6 and P37 declared *B. subtilis* on their labels comprised *B. licheniformis*. *B. sphaericus* was detected in four products (P1, P12, P33, and P34), though was not listed on the product labels. *B. amyloquefaciens* was stated on the label of product P9 and the finding was members of the *B. subtilis* cluster. Product P24 and P25 declared *B. cereus* var. toyoi, and other *Bacillus* species were detected. *L. rhamnosus* and *L. casei*-group (*L. casei* and *L. paracasei*) were found in product P7 but these species were not listed on the label. Four products (P7, P12, P31, and P32) stated *L. acidophilus* on their product label, but none was found. Products P6, P31, and P32 were labeled *S. faecium*. Three products (P41, P44, and P45) were vaguely labelled, particularly lactic acid bacteria without specific species. Based on the PCR results, 41 products were not positive for *Clostridium*, except 4 products (P15, P16, P17, and P42) which were formulated with only *Clostridium butyricum*.

### 2.2. Contamination of E. coli and Salmonella in Whole Probiotic Products (n = 45)

None of the probiotic products tested (*n* = 45) were positive for *E. coli* and *Salmonella*.

### 2.3. Phenotypic AMR in the Bacterial Isolates (n = 64) from Probiotic Products

The MICs of 14 antimicrobials were analyzed in 64 probiotic bacterial isolates including *Bacillus* (*n* = 54), *Enterococcus* (*n* = 4), and *Lactobacillus* (*n* = 6). Thirty-three isolates (51.6%) were resistant to at least one antimicrobial agent. Overall, the resistance to chloramphenicol (20.3%) was highest among probiotic bacteria, followed by resistance to trimethoprim (17.2%), clindamycin (15.6%), sulfamethoxazole (15.6%), erythromycin (9.4%), vancomycin (9.4%), ampicillin (7.8%), tetracycline (7.8%), ciprofloxacin (6.3%), streptomycin (4.7%), and kanamycin (4.7%). All isolates were sensitive to gentamycin, meropenem, and rifampicin. The distribution of MICs of bacterial isolates from probiotic products is shown Table 2.

Among the *Bacillus* isolates, all *B. subtilis* (*n* = 8) and members of the *B. subtilis* cluster (*n* = 23) were phenotypically susceptible to all antimicrobials. All *B. licheniformis* isolates (*n* = 9) exhibited resistance to chloramphenicol and clindamycin. Five *B. licheniformis* isolates showed high resistance to erythromycin with MIC > 128 µg/mL. Of the four *B. sphaericus* isolates, one was resistant to chloramphenicol, erythromycin, and clindamycin, followed by three others which were resistant to trimethoprim and sulfamethoxazole. Two isolates of other *Bacillus* species were resistant to chloramphenicol, tetracycline, trimethoprim, and sulfamethoxazole. Four *Enterococcus* isolates were sensitive to all antimicrobials tested, except sulfamethoxazole. In terms of the six *Lactobacillus* isolates, only *L. rhamnosus* isolate was susceptible to all antimicrobials, except vancomycin. The other five isolates showed resistance to ampicillin, streptomycin, kanamycin, chloramphenicol, tetracycline, trimethoprim, ciprofloxacin, and vancomycin.

### 2.4. Presence of AMR Genes in Whole Probiotic Products (n = 45)

In total, 16 of the 45 products (35.5%) were positive for at least one AMR gene (Table 1). Fourteen products had 1–7 AMR genes. Two products (P12 and P43) contained 12 and 13 AMR genes, respectively. The genes encoding resistance to aminoglycoside (*ant(4′)-Ia*), quinolone (*oqxAB*), and sulfonamide (*sul1*) were found most frequently, being present in 6/45 (13.3%) products. All positive PCR products were correctly confirmed through sequencing. AMR phenotypes in most bacterial isolates were not correlated with AMR genes found in probiotic products.

### 2.5. Transfer of AMR Genes

The conjugation experiments showed that only three *Lactobacillus* isolates, including one *L. delbrueckii* and two other *Lactobacillus* species, could horizontally transfer streptomycin resistance to the *E. coli* recipients. The MICs of streptomycin for transconjugants increased more than four-fold, from 4 µg/mL to 512 µg/mL. No new transconjugants were found to contain streptomycin resistance in the genes tested.

## 3. Discussion

One of the major findings in this study was the inadequate quality and the presence of AMR genes in probiotics commercially available for food animals.

A total of 21 products were collected from original bags or bottles, whereas 24 others were collected from opened bags in feed mills or farms. The probiotics collected from opened bags could be implicated with several problems such as a decrease in microbiological quality, contamination by other bacteria or AMR determinants during manufacturing or unsuitable storage conditions.

Numerous (*n* = 29) probiotic products showed microbiological quality deficiencies in this study, particularly low viable cells (*n* = 11), the misidentification of bacterial species (*n* = 26), and inadequate descriptions of label contents (*n* = 3). The health benefits of probiotics depend on the number of viable organisms; therefore, any decrease may negatively implicate the effectiveness of probiotics. The low number of viable cells could be due to poor quality control at some stages of production, including the drying process, strain-dependent loss of viability, and packaging, and storage conditions, in agreement with previous studies in probiotics used for humans and animals [4,11]. Additional factors during sample handling may influence the discrepancies observed, e.g., the representativeness of the samples collected, the homogeneity of the samples, etc. Probiotics in powder form may cause problems with uniform distribution. However, both the powdered and liquid probiotic products included in this study were carefully mixed before withdrawing analytical samples. It was suggested that a minimum of 10 g of dry samples should be used to ensure the representativeness of the test sample [12]. In this study, 20 g of each powdered sample was tested in duplicate. Therefore, this covered an uncertainty of 5% and satisfied the recommendation.

The probiotic effects are different among bacterial strains and species. Different strains of the same species produce different health beneficial effects, so the label should specify the strains of species included. However, the present study revealed that several products were mislabeled at the bacterial species level and no organisms were identified at strain level. In addition, some vague descriptions of label contents did not contain bacterial names such as lactic acid bacteria. Previous studies showed that many products comprised other *Bacillus* species misidentified as *B. subtilis* [4], similar to the results of the present findings. *B. subtiblis* was the most common misidentified species. The *B. subtilis* cluster, *B. licheniformis*, *B. sphaercius*, and other *Bacillus* species were labeled as *B. subtilis*. Such mislabeling of bacterial species was possibly the use of unreliable identification methods by producers. Due to the limitations of the differentiation ability of ARDRA, *B. amyloliquefaciens* and *B. cereus* var. toyoi could not be confirmed, so the products (P9, P24, and P25) claiming to contain these species could not be defined as having been mislabelled. *S. faecium* was reclassified as *E. faecium* in 1984 [13]; however, mislabeling *E. faecium* as *S. faecium* was still common. Such inadequate labelling certainly raises concerns about the producers’ inattention to detail, which does not provide much confidence in using probiotics. The use of *Enterococcus* and *Clostridium* strains as probiotics has recently gained popularity; however, none of them are considered as generally safe (GRAS) or QPS due to their association with human illnesses, virulence factors, and AMR genes [8]. Thus, the manufacturers should scrutinize them before using them as probiotics.

Undetected foodborne pathogens, *Salmonella* and *E. coli*, could be due to no contamination of pathogenic bacteria in raw materials and unsuitable conditions for growth (e.g., the dryness of probiotic products). The presence of pathogenic bacteria should be controlled during the probiotic production process because of their ability to carry and transfer AMR genes inter- and intra-species. The latter may lead to the spread of AMR bacteria and their determinants in livestock.

The *Lactobacillus* and *Bacillus* isolates generally showed resistance to a broad range of antibiotics, as previously observed [4,14]. Antimicrobial susceptibility among *Lactobacillus* and *Bacillus* isolates was species-specific. *B. licheniformis* isolates’ high resistance to chloramphenicol and clindamycin was attributed to the intrinsic resistance characteristics of this species due to the uniform distributions of the MIC values [15]. In contrast, the *B. subtilis* and members of the *B. subtilis* cluster were completely sensitive to all antimicrobials tested. Another study showed that the *B. subtilis* used for oral bacteriotherapy was resistant to chloramphenicol, tetracycline, rifampicin, and streptomycin [16]. *Lactobacillus* species have possessed various intrinsic mechanisms of resistance to aminoglycosides (neomycin, kanamycin, streptomycin, and gentamicin), glycopeptides (vancomycin), nucleic acid synthesis inhibitors (quinolones and fluroquinolones), and folic acid inhibitors (trimethoprim and sulfamethoxazole) [17]. However, *Lactobacillus* strains often contain plasmids that play a role in the dissemination of AMR genes [4,5,6]. In this study, streptomycin resistance determinants could be transferred from *Lactobacillus* to *E. coli* via conjugation but none of the AMR genes were detected. This means these species may carry other streptomycin resistance genes that were not examined in this study. Although *E. feacium* isolates in this study were susceptible to almost clinically important antimicrobials, the use of *Enterococcus* for probiotics should be considered due to the rise in nosocomial infections with vancomycin-resistant enterococci (VRE) [18].

As per the EFSA guidelines, the assessment of a candidate microorganism for QPS status necessitates a thorough examination of the AMR determinants and their potential mobility. Notably, β-lactamase genes (*bla*_OXA-1-like_ and *bla*_SHV_) and plasmid-mediated quinolone resistance genes (PMQR) are prevalent in Enterobacteriaceae isolated from food animals but infrequently detected in Gram-positive bacteria [19,20,21,22]. Our investigation revealed the consistent presence of several aminoglycoside genes in Gram-positive bacteria, such as *aadE*, *aac(3)-II*, *aac(6′)-aph(2”)*, *aph(3′)-IIIa*, and *ant(4′)-Ia*. Conversely, some genes, including *aadA1*, *aadA2*, and *strA-strB*, were identified in both Gram-positive and Gram-negative bacteria [6,23,24].

Despite stringent regulations on aminoglycoside use and the prohibition of chloramphenicol in animal husbandry, the encoding of these antimicrobials in bacterial species may result from co-selection with other genes in the same mobile genetic element. For instance, the close association of *cmlA* with other gene cassettes (*aadA1* and *aadA2*) within class 1 integrons underscores the chloramphenicol co-selection in the absence of selective pressure. Various genes, such as *sul1* and *dfr*, commonly associated with class 1 integrons and gene cassettes and facilitating the dissemination of MDR determinants [25] *tetA* and *tetB*, have been previously detected in *Salmonella* and *E. coli* from diverse sources [26,27], while *tetM* and *tetL* are prevalent in Gram-positive bacteria [5]. The *mef(A)* gene exhibits predominance in both Gram-negative and Gram-positive bacteria [28,29]. Notably, *vanC* demonstrates intrinsic, non-transferable, low-level resistance to vancomycin [18]. The identification of AMR genes in imported products raises concerns, indicating a global circulation of these genes during trade. Possible origins include the microorganism sources used in probiotic production and contamination during manufacturing or storage, particularly in products from opened bags at feed mills or farms.

The uncorrelation between AMR phenotypes of bacterial isolates and AMR genotypes of probiotic products may be possibly explained by the following reasons. First, AMR genes could be derived from other bacteria-contaminated probiotic products. Second, AMR genes conferring resistance to bacterial isolates were not tested in this study. Last, probiotic strains may carry AMR genes, but the resistance phenotypes cannot be expressed. Therefore, AMR genes should be systemically tested in not only probiotic strains but also probiotic products before launching to the market to provide an overview of sources of AMR genes in order to have a timely intervention.

Several issues of this study should be considered when interpreting the results. This study is qualitative, not quantitative, therefore the presence of transferable AMR genes in probiotics was detected, but the amount of genetic material per dose of probiotic was not quantified. A quantitative study should be carried out for the risk assessment of AMR transmission. The presence of AMR genes in bacterial isolates was not investigated in this study due to it not being a requirement for the transfer of genetic material. Additional studies for microorganisms harboring certain AMR genes are necessary to evaluate the correspondence between the genotypic AMR and phenotypic resistance in bacterial isolates.

## 4. Materials and Methods

### 4.1. Sample Collection (n = 45)

A total of 45 commercial probiotic products for food animals, including 2 liquid products (P1 and P7) and 43 powder products, were collected during March 2019–December 2021 (Table 1). All, except for P1, P6, P7, P33, and P34, were imported products. The probiotic product distributors who agreed to participate in the project submitted the products to the laboratory at the Department of Veterinary Public Health, Faculty of Veterinary Science, Chulalongkorn University. Of the 45 products, 24 products were collected from opened bags at feed mills or farms (P23–P42), while the others (*n* = 21) were obtained from original whole packages (P1–P22 and P43–P45). Each sample was mixed to ensure homogeneity before taking portions. At least 100 g or milliliters of each sample were aseptically collected, stored in either lightproof bottles or bags, and submitted to the laboratory within 24 h. All products were kept at room temperature within 7 days of being collected. The samples from the same batch were avoided. All samples tested were at least 3 months before the expiration date. The information declared on the leaflet, including numbers of bacterial cells, bacterial species, and expiry date was collected. An aseptic technique was applied for sample collection and throughout the experiment.

### 4.2. PCR and Nucleotide Sequencing

Whole cell DNA template was prepared from all bacterial isolates using whole cell boiling procedure [30]. DNA from probiotic products was directly extracted using GeneJET^TM^ Genomic DNA Purification Kit (Thermo Scientific, Waltham, MA, USA). All PCR reactions were performed using TopTaq Master Mix (Qiagen, Hilden, Germany) according to manufacturer’s instructions. The primers used in this study are shown in Tables S1 and S2.

### 4.3. Determination of Microbiological Quality (n = 45)

#### 4.3.1. Enumeration, Isolation, and Species Confirmation of *Lactobacillus*, *Bacillus*, and *Enterococcus* (*n* = 41)

Forty-one probiotic products (*n* = 41), except P15, P16, P17, and P44, were examined for number of viable cells including *Lactobacillus*, *Bacillus*, and *Enterococcus*. Prior to the isolation and enumeration of target bacteria, all samples (*n* = 41), either liquid or dried products, were prepared as follows. For dried products, 20 g of each sample was dissolved in 180 mL peptone saline diluting fluid (PSD; peptone 1.0 g and NaCl 8.5 g in 1000 mL distilled water) [12]. For liquid products, 1 mL of each liquid product was diluted in 9 mL PSD. The samples were 10-fold serially diluted and the bacterial count for each product was performed in duplicate.

Enumeration and isolation of *Lactobacillus* was performed by pour plate method using De Man, Rogosa, and Sharpe (MRS) agar (Difco^®^, Sparks, MD, USA) [31]. Briefly, 1 mL of each diluted sample was mixed with 15 mL molten MRS agar in a petri dish and incubated under microaerophilic conditions at 30 °C for 48–72 h. The *Bacillus* and *Enterococcus* were isolated and counted using the spread plate method using Mannitol Egg Yolk Polymyxin (MYP) agar (Difco^®^) [32] and Bile Aesculin agar (BEA) (Oxoid^®^, Hampshire, UK) [33], respectively. A 100 μL diluted sample was spread on MYP agar for *Bacillus* and BEA agar for *Enterococcus* and incubated at 37 °C for 24 h. For each product, the number of bacterial colonies were the means of duplicated counts. Three to five typical colonies of each target bacteria were selected for further identification of species. All isolates were stored in 20% glycerol at −80 °C.

The *Bacillus* (*n* = 190), *Lactobacillus* (*n* = 20), and *Enterococcus* (*n* = 20) isolates were confirmed for their genus and species by PCR. The genus and species of *Bacillus* were identified using Amplified Ribosomal DNA Restriction Analysis (ARDRA) [34]. For *Lactobacillus* and *Enterococcus*, the genus was detected using simplex PCR (Table S1), while the species were confirmed by multiplex PCR (Table S2).

#### 4.3.2. Detection of *Clostridium* (*n* = 45)

The presence of *Clostridium* was detected in DNA from all products (*n* = 45), of which only 4 products (P15, P16, P17, and P44) claimed to contain *Clostridium* species on the labels. Simplex PCR assays were performed to detect genus and species as previously described (Table S1).

#### 4.3.3. Determination of Salmonella and *E. coli* in Whole Probiotic Products (*n* = 45)

All samples (*n* = 45) were examined for the presence of *Salmonella* and *E. coli*. The *Salmonella* strains were isolated using the standard methods described in ISO 6579:2002(en) [35]. All *Salmonella* isolates were serotyped by slide agglutination based on the Kauffmann–White schemes using commercially available antiserum [36]. A single colony of each serotype from each positive sample was collected. The *E. coli* strains were isolated and biochemically confirmed using standard protocols according to the Bacteriological Analytical Manual [37]. One colony from each positive sample was collected.

### 4.4. Determination of AMR Characteristics (n = 45)

#### 4.4.1. Antimicrobial Susceptibility Testing for Probiotic Bacterial Isolates

One isolate of each probiotic species found in each positive sample was collected. A total of 64 probiotic bacterial isolates, including *Bacillus* (*n* = 54), *Lactobacillus* (*n* = 6), and *Enterococcus* (*n* = 4), were examined for their susceptibilities to 14 antimicrobial agents, including ampicillin (AMP), meropenem (MER), streptomycin (STR), kanamycin (KAN), gentamicin (GEN), chloramphenicol (CHL), tetracycline (TET), erythromycin (ERY), vancomycin (VAN), trimethoprim (TRI), sulfamethoxazole (SUL), ciprofloxacin (CIP), clindamycin (CLI), and rifampicin (RIF), by determining the minimum inhibitory concentrations (MICs). All antimicrobial agents were purchased from Sigma-Aldrich^®^ (Steinheim, Germany). MICs of *Lactobacillus* were determined by broth microdilution method using lactic acid bacterial (LAB) susceptibility test medium [38]. For *Bacillus* and *Enterococcus*, the determination of MICs was performed using a two-fold agar dilution method [39]. The interpretive criteria used are shown in Table 2. Priority was given to clinical breakpoints according to Clinical and Laboratory Standards Institute (CLSI) and European Committee on Antimicrobial Susceptibility Testing (EUCAST) [40,41]. When the breakpoints were not available, the epidemiology cut-off (ECOFF) values, according to EUCAST and EFSA Panel, were used [41,42]. It should be noted that clinical breakpoints are used to identify “clinical resistance” where isolates are not inhibited by antibiotics with standard dosing regimens and normal dosage schedules. ECOFFs serve to define “microbial resistance” where isolates are defined as non-wild type by the presence of an acquired or mutational antibiotic resistance mechanism and does not necessarily result in treatment failure. Sources of interpretive criteria used are as follows: *Lactobacillus*, meropenem/sulfamethozole—CBP [40], ampicillin/streptomycin/kanamycin/gentamicin/chloramphenicol/tetracycline/erythromycin/vancomycin/clindamycin/trimethoprim/ciprofloxacin/rifampicin—EFSA [42], *Enterococcus*, sulfamethoxazole—CBE [40], ampicillin/streptomycin/kanamycin/gentamicin/chloramphenicol/tetracycline/erythromycin/vancomycin/clindamycin—EUCAST [41], and meropenem/ciprofloxacin/trimethoprim/rifampicin—EFSA [42]; and *Bacillus*, meropenem/sulfamethoxazole—CBP [40], streptomycin/kanamycin/gentamicin/chloramphenicol/tetracycline/erythromycin/vancomycin/clindamycin—EUCAST [41], and ampicillin/trimethoprim/ciprofloxacin/rifampicin—EFSA [42]. *E. coli* ATCC 25922, *Staphylococcus aureus* ATCC 29213, and *Enterococcus faecalis* ATCC 29212 were used as quality control.

#### 4.4.2. Detection of AMR Genes in Probiotic Products (*n* = 45)

The presence of 112 genes encoding resistance to clinically important antibiotics was screened in whole probiotic products (*n* = 45). The PCR products of all positive samples were submitted for nucleotide sequencing at First Base Laboratories (Selangor Darul Ehsan, Malaysia). The DNA sequencing results were compared to reference sequences published on GeneBank Database using BLAST algorithm (www.ncbi.nlm.nih.gov, accessed on 26 January 2024).

#### 4.4.3. Conjugation Experiments

The *Lactobacillus* (*n* = 5) and *Bacillus* (*n* = 17) isolates exhibiting resistance phenotype were selected to test the transferability of AMR genes using the biparental mating method [43]. Horizontal transfer of chloramphenicol, tetracycline, trimethoprim, and clindamycin resistance was tested for *Bacillus*, while that of ampicillin, streptomycin, kanamycin, chloramphenicol, tetracycline, and ciprofloxacin resistance was examined for *Lactobacillus*. The spontaneous rifampicin-resistant *E. coli* K12 strain MG1655 (MIC = 256 µg/mL) was used as recipient. Non-selective media used for filter mating were Luria-Bertani (LB) media and Brain Heart Infusion (BHI) media (Difco^®^, Sparks, MD, USA) for *Bacillus* and *Lactobacillus*, respectively. The *E. coli* transconjugants were confirmed on Eosin Methylene Blue (EMB) agar (Difco^®^) containing ampicillin (100 µg/mL), streptomycin (50 µg/mL), kanamycin (35 µg/mL), chloramphenicol (25 µg/mL), tetracycline (10 µg/mL), trimethoprim (100 µg/mL), and ciprofloxacin (0.064 µg/mL). All transconjugants were examined for their MICs for all antibiotics, as mentioned above. The transconjugants with a four-fold MIC increase compared to the recipients were tested for AMR determinants from donors using PCR, as described above.

## 5. Conclusions

The probiotic products tested in this study were qualitatively or quantitatively unsatisfactory as claimed on the labels. Inadequate label descriptions were observed, including spelling errors and none-specific strains. The antimicrobial resistance among probiotic isolates appeared to vary among species. Streptomycin resistances were transferred horizontally, but no resistant streptomycin was detected. Most of the AMR genes detected in this study are transferable and have been frequently observed in either Gram-positive or Gram-negative bacteria or both. The findings can be used to support improvements in the regulation of probiotic products by the relevant authorities. The manufacturers and producers should have developed policies to control the quality of their probiotic products. They should quarantine the number of viable organisms declared until the expired date. The genus, species, and strains of probiotic organisms should be accurately identified. Only nontoxic and nonpathogenic strains that do not harbor AMR determinants can be used for probiotic products. The farmers and other food-animal producers should wisely choose the probiotic products that are approved to be sold on the market by the relevant authorities. The data obtained can be also used as part of AMR risk assessment in probiotic products.

## Figures and Tables

**Table 1:** Comparison of information given on labels and the observation in this study (*n* = 45).

Product	Labelling Information		Observation in This Study					
	Strains	Number ^a^	Strains	Number	Strain ID	Specific Species	AMR Patterns	AMR Genes
P1	*B. licheniformis* *B. subtilis*	1.9 × 10^11^	*Bacillus* spp.	2.14 × 10^9^	B1.1	*B. subtilis*	-	
					B1.3	Members of the *B. subtilis* cluster ^b^	-	
					B1.5	*B. sphaericus*	CHL-CLI-ERY	
P2	*B. subtilis*	1.48 × 10^11^	*Bacillus* spp.	9.2 × 10^10^	B2.1	*B. subtilis*	*-*	
					B2.2	Members of the *B. subtilis* cluster	*-*	
					B2.3	Other *Bacillus* species	*-*	
P3	*B. licheniformis* *B. subtilis*	10.04 × 10^10^ 4.76 × 10^10^	*Bacillus* spp.	9.4 × 10^10^	B3.1	*B. subtilis*	-	*oqxAB*
					B3.3	Members of the *B. subtilis* cluster	-	
P4	*B. subtilis*	4 × 10^11^	*Bacillus* spp.	7.65 × 10^11^	B4.1	Members of the *B. subtilis* cluster	-	*oqxAB*, *aadA2*, *sul1*
P5	*B. subtilis*	4 × 10^11^	*Bacillus* spp.	7.2 × 10^12^	B5.1	Members of the *B. subtilis* cluster	-	*sul1*
P6	*B. subtilis* *S. faecium*	5 × 10^9^ 5 × 10^9^	*Bacillus* spp. *Enterococcus* spp.	7.2 × 10^10^ 9.2 × 10^11^	B6.1	*B. licheniformis*	CHL-CLI-ERY	*qnrD*, *tetA*, *tetM*
					E6.1	*E. faecium*	SUL	
P7	*L. acidophilus* *L. plantarum* *B. subtilis* *B. licheniformis*	1 × 10^9^ 1 × 10^9^ 1 × 10^9^ 1 × 10^9^	*Lactobacillus* spp. *Bacillus* spp.	1.88 × 10^9^ 8.4 × 10^13^	B7.1	*L. plantarum*	AMP-CIP-TRI-VAN	
					L7.1	*L. rhamnosus*	VAN	
					L7.2	*L. casei*-group ^c^	AMP-CHL-TRI-VAN	
					L7.4.	Members of the *B. subtilis* cluster		
P8	*E. faecium*	8.4 × 10^11^	*Enterococcus* spp.	3.85 × 10^15^	E8.1	*E. faecium*	SUL	
P9	*B. amyloliquefaciens*	1 × 10^13^	*Bacillus* spp.	2.01 × 10^13^	B9.1	Members of the *B. subtilis* cluster	-	
P10	*B. subtilis*	1 × 10^13^	*Bacillus* spp.	6 × 10^13^	B10.1	Members of the *B. subtilis* cluster	-	
P11	*B. licheniformis*	1.6 × 10^12^	*Bacillus* spp.	5.6 × 10^12^	B11.1	*B. licheniformis*	CHL-CLI-ERY	
P12	*B. coagulans* *B. subtilis* *L. acidophilus*	1.5 × 10^12^ 1 × 10^12^ 1.5 × 10^12^	*Bacillus* spp.	1.31 × 10^7^	B12.1	Members of the *B. subtilis* cluster	-	*bla*_OXA-1-like_, *aac(6′)-Ib-cr*, *qnrB*, *aadA1*, *aadA2*, *strA-strB*, *aac(3)-II*, *tetA*, *catA*, *dfrA14*, *sul1*
					B12.2	*B. sphaericus*	SUL-TRI	
P13	*B. licheniformis* *B. subtilis*	2.56 × 10^11^	*Bacillus* spp.	2.3 × 10^11^	B13.1	*B. licheniformis*	CHL-CLI-ERY	*strA-strB*, *aadE*, *tetM*
					B13.3	*B. subtilis*	-	
P14	*E. faecium*	5 × 10^14^	*Enterococcus* spp.	1.85 × 10^14^	E14.1	*E. faecium*	SUL	
P15	*C. butyricum*	1.25 × 10^12^	*Clostridium* spp.	NT	-	*C. butyricum*	NT	
P16	*C. butyricum*	5 × 10^8^	*Clostridium* spp.	NT	-	*C. butyricum*	NT	
P17	*C. butyricum*	5 × 10^8^	*Clostridium* spp.	NT	-	*C. butyricum*	NT	
P18	*B. licheniformis*	1.6 × 10^13^	*Bacillus* spp.	8.4 × 10^13^	B18.1	*B. licheniformis*	CHL-CLI	
P19	*B. subtilis*	1 × 10^13^	*Bacillus* spp.	4.9 × 10^13^	B19.1	Other *Bacillus* species	*-*	
P20	*B. subtilis*	1 × 10^13^	*Bacillus* spp.	2.15 × 10^13^	B20.1	*B. subtilis*	*-*	
P21	*B. subtilis*	1 × 10^13^	*Bacillus* spp.	3.7 × 10^13^	B21.1	*B. subtilis*	*-*	
P22	*B. subtilis*	1 × 10^12^	*Bacillus* spp.	3.2 × 10^12^	B22.1	Members of the *B. subtilis* cluster	*-*	
P23	*B. subtilis*	1 × 10^12^	*Bacillus* spp.	5.05 × 10^12^	B23.1	Members of the *B. subtilis* cluster	*-*	
P24	*B. cereus toyoi*	1 × 10^13^	*Bacillus* spp.	6.3 × 10^12^	B24.1	Other *Bacillus* species	CHL-SUL-TET-TRI	
P25	*B. cereus toyoi*	1 × 10^13^	*Bacillus* spp.	2.85 × 10^12^	B25.1	Other *Bacillus* species	CHL-SUL-TET-TRI	
P26	*B. licheniformis*	3.2 × 10^12^	*Bacillus* spp.	3.7 × 10^12^	B26.1	*B. licheniformis*	CHL-CLI-ERY	
P27	*B. subtilis*	1.48 × 10^11^	*Bacillus* spp.	8.3 × 10^10^	B27.1	*B. subtilis*	-	
					B27.1	Members of the *B. subtilis* cluster	-	
P28	*B. subtilis*	7.5 × 10^10^	*Bacillus* spp.	4.55 × 10^10^	B28.1	Members of the *B. subtilis* cluster	-	
					B28.5	Other *Bacillus* species	-	
P29	*B. subtilis*	7.5 × 10^10^	*Bacillus* spp.	3.85 × 10^10^	B29.1	Other *Bacillus* species	-	
P30	*B. subtilis* *B. licheniformis*	1.48 × 10^11^	*Bacillus* spp.	5.35 × 10^10^	B30.2	*B. subtilis*	-	
					B30.4	Members of the *B. subtilis* cluster	-	
					B30.5	Other *Bacillus* species	-	
P31	*B. subtilis* *B. licheniformis* *L. acidophilus* *L. casei* *S. faecium*	6.5 × 10^10^ 5.8 × 10^10^ 6 × 10^9^ 1 × 10^9^ 1.5 × 10^9^	*Bacillus* spp.	1.93 × 10^10^	B31.1	Members of the *B. subtilis* cluster	-	*ant(4′)-Ia*
					B31.4	Other *Bacillus* species	-	
P32	*B. subtilis* *B. licheniformis* *L. acidophilus* *L. casei* *S. faecium*	6.5 × 10^10^ 5.8 × 10^10^ 6 × 10^9^ 1 × 10^9^ 1.5 × 10^9^	*Bacillus* spp.	2.45 × 10^10^	B32.1	Members of the *B. subtilis* cluster	-	*ant(4′)-Ia*, *mefA*
					B32.4	Other *Bacillus* species	-	
P33	*B. subtilis*	4.7 × 10^8^	*Bacillus* spp.	3.25 × 10^10^	*B33.1*	*B. sphaericus*	SUL-TRI	
					B33.3	Members of *B. subtilis* cluster	-	
					B33.4	Other *Bacillus* species	SUL-TRI	
P34	*B. subtilis*	4.7 × 10^8^	*Bacillus* spp.	1.65 × 10^8^	B34.1	*B. sphaericus*	SUL-TRI	
P35	*B. subtilis*	2 × 10^11^	*Bacillus* spp.	3.9 × 10^11^	B35.1	Members of the *B. subtilis* cluster	-	
P36	*B. subtilis*	2 × 10^11^	*Bacillus* spp.	4.65 × 10^11^	B36.1	Members of the *B. subtilis* cluster	-	
P37	*B. subtilis*	2 × 10^11^	*Bacillus* spp.	3.3 × 10^11^	B37.1	*B. licheniformis*	CHL-CLI-ERY	
					B37.3	Members of the *B. subtilis* cluster	-	
P38	*B. licheniformis*	3.2 × 10^12^	*Bacillus* spp.	1.8 × 10^12^	B38.1	*B. licheniformis*	CHL-CLI	*oqxAB*, *ant(4′)-Ia*, *catA*, *mefA*
P39	*B. licheniformis*	3.2 × 10^12^	*Bacillus* spp.	1.7 × 10^12^	B39.1	*B. licheniformis*	CHL-CLI	*ant(4′)-Ia*, *aac(6′)-aph(2″)*, *catA*, *mefA*
P40	*B. licheniformis*	3.2 × 10^12^	*Bacillus* spp.	2.45 × 10^12^	B40.1	*B. licheniformis*	CHL-CLI	*ant(4′)-Ia*, *aph(3′)-IIIa*, *catA*
P41	Lactic acid bacteria	1.34 × 10^12^	*Lactobacillus* spp.	2.7 × 10^11^	L41.1	*L. delbrueckii*	AMP-CIP-KAN-STR-TET-TRI-VAN	*tetM*, *tetL*
						Other lactic acid species	-	
P42	*C. butyricum*	5 × 10^8^	*Clostridium* spp.	NT	-	*C. butyricum*	NT	*qnrS*, *qnrD*, *ant(4′)-Ia*, *mefA*
P43	*B. licheniformis* *B. subtilis* *B. pumilus* *E. faecium* *E. faecalis*	≥1 × 10^12^ ≥1 × 10^11^	*Bacillus* spp. *Enterococcus* spp.	2.12 × 10^12^ 1.54 × 10^11^	B43.1	Members of the *B. subtilis* cluster	-	*bla*_SHV,_, *oqxAB*, *qnrS*, *aadA2*, *strA-strB*, *aac(3)-II*, *tetA*, *tetB*, *dfrA12*, *dfrA14*, *sul1*, *vanC*
					E43.1	*E. faecium*	SUL	
P44	Lactic acid bacteria *B. subtilis*	≥7 × 10^12^ ≥3 × 10^12^	*Lactobacillus* spp. *Bacillus* spp.	8.1 × 10^11^ 7.35 × 10^12^	B44.1	Other *Lactobacillus* species,	AMP-CIP-KAN-STR-TET-TRI-VAN	*oqxAB*, *aac(6′)-Ib-cr*, *aadA2*, *aac(6′)-aph(2″)*, *dfrA12*, *sul1*
					L44.1	Members of the *B. subtilis* cluster	-	
P45	Lactic acid bacteria *B. subtilis*	≥7 × 10^12^ ≥3 × 10^12^	*Lactobacillus* spp. *Bacillus* spp.	1.85 × 10^12^ 9.8 × 10^12^	B45.1	Other *Lactobacillus* species	AMP-CIP-KAN-STR-TET-TRI-VAN	*oqxAB*, *aac(6′)-Ib-cr*, *aadA2*, *aac(6′)-aph(2″)*, *dfrA12*, *sul1*
					L45.1	Members of the *B. subtilis* cluster	-	

^a^ Unit is Colony Forming Unit (CFU)/kg for all products except for product P1 and P7 (CFU/L). ^b^ *B. pumilus*, *B. amyloliquefaciens*, and *B. atropheus.* ^c^ *L. casei* and *L. paracasei*.—isolates were susceptible to all antimicrobials tested. NT, not tested. Bold letters indicate antimicrobial resistance (AMR) genes corresponding to AMR phenotypes of bacterial isolates. Abbreviations: AMP, ampicillin; STR, streptomycin; KAN, kanamycin; CHL, chloramphenicol; TET, tetracycline; ERY, erythromycin; TRI, trimethoprim; SUL, sulfamethoxazole; CIP, ciprofloxacin; VAN, vancomycin; CLI, clindamycin.

**Table 2:** Distribution of MICs of bacterial isolates from probiotic products (*n* = 64).

Strain (*n*)	Distribution of MICs (μg/mL)														No. of Resistant Isolates	Distribution of MICs (μg/mL)														No. of Resistant Isolates
	≤0.125	0.25	0.5	1	2	4	8	16	32	64	128	256	512	≥1024		≤0.125	0.25	0.5	1	2	4	8	16	32	64	128	256	512	≥1024	
	AMP															CHL														
*B. subtilis* (8)	8														0						7	1								0
*B. licheniformis* (9)	8	1													0								8	1						9
*B. sphaericus* (4)	3				1										0						2	1	1							1
Other *Bacillus* spp. (10)	10														0					3	5				2					2
Members of *B. subtilis* cluster (23)	23														0			1	5		17									0
*E. faecium* (4)				4											0					4										0
*L. casei*-group (1)							1								1							1								1
*L. plantarum* (1)							1								1							1								0
*L. rhamnosus* (1)			1												0						1									0
*L. delbrueckii* (1)					1										1						1									0
Other *Lactobacillus* species (2)						2									2						2									0
Subtotal	52	1	1	4	2	2	2	0	0	0	0	0	0	0	5	0	0	1	5	7	35	4	9	1	2	0	0	0	0	13
	CIP															CLI														
*B. subtilis* (8)	8														0	2	1	3	2											0
*B. licheniformis* (9)	9														0								3	5	1					9
*B. sphaericus* (4)	4														0			2	1			1								1
Other *Bacillus* spp. (10)	10														0	3	4	3												0
Members of *B. subtilis* cluster (23)	23														0	2	13	7	0	1										0
*E. faecium* (4)		4													0			1		2	1									0
*L. casei*-group (1)					1										0															0
*L. plantarum* (1)								1							1		1													0
*L. rhamnosus* (1)				1											0															0
*L. delbrueckii* (1)									1						1															0
Other *Lactobacillus* species (2)										2					2															0
Subtotal	54	4	0	1	1	0	0	1	1	2	0	0	0	0	4	7	19	16	3	3	1	1	3	5	1	0	0	0	0	10
	ERY															GEN														
*B. subtilis* (8)		8													0	8														0
*B. licheniformis* (9)		4									5				5	5	4													0
*B. sphaericus* (4)		3									1				1	3	1													0
Other *Bacillus* spp. (10)		7	1	2											0	8	2													0
Members of *B. subtilis* cluster (23)		22	1												0		23													0
*E. faecium* (4)	4														0						4									0
*L. casei*-group (1)	1														0					1										0
*L. plantarum* (1)	1														0						1									0
*L. rhamnosus* (1)	1														0					1										0
*L. delbrueckii* (1)	1														0					1										0
Other *Lactobacillus* species (2)	2														0					1	1									0
Subtotal	10	44	2	2	0	0	0	0	0	0	6	0	0	0	6	24	30	0	0	4	6	0	0	0	0	0	0	0	0	0
	KAN															MER														
*B. subtilis* (8)				8											0	8														0
*B. licheniformis* (9)				9											0	9														0
*B. sphaericus* (4)				4											0	4														0
Other *Bacillus* spp. (10)				10											0	10														0
Members of *B. subtilis* cluster (23)				23											0	23														0
*E. faecium* (4)													4		0						3	1								0
*L. casei*-group (1)										1					0	1														0
*L. plantarum* (1)										1					0		1													0
*L. rhamnosus* (1)										1					0					1										0
*L. delbrueckii* (1)											1				1	1														0
Other *Lactobacillus* species (2)									1		1				2			2												0
Subtotal	0	0	0	54	0	0	0	0	1	3	2	0	4	0	3	56	1	2	0	1	3	1	0	0	0	0	0	0	0	0
	RIF															STR														
*B. subtilis* (8)	8														0				8											0
*B. licheniformis* (9)	9														0				9											0
*B. sphaericus* (4)	4														0				3		1									0
Other *Bacillus* spp. (10)	10														0				7	3										0
Members of *B. subtilis* cluster (23)	23														0				22	1										0
*E. faecium* (4)	4														0				4											0
*L. casei*-group (1)							1								0								1							0
*L. plantarum* (1)			1												0									1						0
*L. rhamnosus* (1)			1												0									1						0
*L. delbrueckii* (1)			1												0											1				1
Other *Lactobacillus* species (2)			2												0									1	1					2
Subtotal	58	0	5	0	0	0	1	0	0	0	0	0	0	0	0	0	0	0	53	4	1	0	1	3	1	1	0	0	0	3
	SUL															TET														
*B. subtilis* (8)			6	2											0	4					3	1								0
*B. licheniformis* (9)			6	1	1	1									0	5				2	1	1								0
*B. sphaericus* (4)				1										3	3	3	1													0
Other *Bacillus* spp. (10)			2	1		3		1						3	3	2	1				3	2			2					2
Members of *B. subtilis* cluster (23)			1	6	4	12									0	6				2	3	12								0
*E. faecium* (4)														4	4	4														0
*L. casei*-group (1)											1				0				1											0
*L. plantarum* (1)											1				0								1							0
*L. rhamnosus* (1)											1				0				1											0
*L. delbrueckii* (1)										1					0												1			1
Other *Lactobacillus* species (2)											2				0									2						2
Subtotal	0	0	15	11	5	16	0	1	0	1	5	0	0	10	10	24	2	0	2	4	10	16	1	2	2	0	1	0	0	5
	TRI															VAN														
*B. subtilis* (8)	8														0		8													0
*B. licheniformis* (9)	9														0		9													0
*B. sphaericus* (4)	1										1	2			3		3	1												0
Other *Bacillus* spp. (10)	3	2	2									3			3		6	1	2		1									0
Members of *B. subtilis* cluster (23)	18	3	1	1											0		22	1												0
*E. faecium* (4)	4														0			4												0
*L. casei*-group (1)									1						1												1			1
*L. plantarum* (1)													1		1												1			1
*L. rhamnosus* (1)								1							0												1			1
*L. delbrueckii* (1)												1			1												1			1
Other *Lactobacillus* species (2)										1			1		2												2			2
Subtotal	43	5	3	1	0	0	0	1	1	1	1	6	2	0	11	0	48	7	2	0	1	0	0	0	0	0	6	0	0	6

Vertical line, breakpoints. As the clinical breakpoints of sulfamethoxazole for *Lactobacillus*, *Bacillus*, and *Enterococcus* are not available, and these species are mainly fastidious Gram-positive bacteria like *Staphylococcus*, therefore the breakpoint of ≥512 µg/mL for *S. aureus* was applied as interpretative criteria [10]. Abbreviation: AMP, ampicillin; STR, streptomycin; KAN, kanamycin; GEN, gentamicin; CHL, chloramphenicol; TET, tetracycline; ERY, erythromycin; TRI, trimethoprim; SUL, sulfamethoxazole; CIP, ciprofloxacin; VAN, vancomycin; CLI, clindamycin; MIC, Minimum Inhibitory Concentration; MER, meropenem; RIF, rifampicin. White fields indicate concentration range of serial dilutions for each antimicrobial.

## Data Availability

Data are contained within the article and Supplementary Materials.

## Supplementary Materials

The following supporting information can be downloaded at: https://www.mdpi.com/article/10.3390/antibiotics13020148/s1, Table S1: Primers used for determination of genus and species of probiotic bacteria; Table S2: Primers used for detection of AMR genes. References [14,19,20,21,22,26,28,34,44,45,46,47,48,49,50,51,52,53,54,55,56,57,58,59,60,61,62,63,64,65,66,67,68,69,70,71,72,73,74,75,76,77,78,79,80,81,82,83,84,85,86,87] are cited in the Supplementary Materials.
