# Supplementary material for: Microbiological Quality and Antimicrobial Resistance of Commercial Probiotic Products for Food-Producing Animals
Source: Antibiotics (Basel). 2024 Feb 1;13(2):148. doi: 10.3390/antibiotics13020148 (PMC10885956; doi:10.3390/antibiotics13020148)
Supplement: Supplementary file 1 [file antibiotics-13-00148-s001.zip › antibiotics-2809014-supplementary.pdf]

antibiotics

MDPI

Article

Microbiological Quality and Antimicrobial Resistance of Commercial Probiotic Products for Food-Producing Animals

Hoang My Tran [1], Rangsiya Prathan [2,3], Si Thu Hein [2,3] and Rungtip Chuanchuen [2,3,*]

1 The International Graduate Course of Veterinary Science and Technology (VST), Faculty of Veterinary Science, Chulalongkorn University, Bangkok 10330, Thailand; hoangmy.bsty@gmail.com
2 Research Unit in Microbial Food Safety and Antimicrobial Resistance, Department of Veterinary Public Health, Faculty of Veterinary Science, Chulalongkorn University, Bangkok 10330, Thailand; rochun.30@gmail.com (R.P.); sithuhein5343@gmail.com (S.T.H.)
3 Center for Antimicrobial Resistance Monitoring in Foodborne Pathogens (in Cooperation with WHO), Faculty of Veterinary Science, Chulalongkorn University, Bangkok 10330, Thailand
* Correspondence: chuanchuen.r@gmail.com

Supplementary Materials

Table S1. Primers used for determination of genus and species of probiotic bacteria.

Primers	Sequence (5′–3′)	PCR Type	PCR Product (bp)	References
Bacillus				
B-K1/F	TCACCAAGGCRACGATGCG	All *Bacillus*	~1114	[34]
B-K1/R1	CGTATTCACCGCGGCATG			
Lactobacillus				
R16-1	CTTGTACACACCGCCCGTCA	Genus-specificity	Variable	[44]
LbLMA1-rev	CTCAAAACTAAACAAAGTTTC	Genus-specificity		[45]
IDL03R	CCACCTTCCTCCGGTTTGTCA	All *Lactobacillus*	-	[46]
IDL04F	AGGGTGAAGTCGTAACAAGTAGCC	All *Lactobacillus*	-	[46]
IDL11F	TGGTCGGCAGAGTAACTGTTGTCG	*L. casei*-group	727	[46]
IDL22R	AACTATCGCTTACGCTACCACTTTGC	*L. acidophilus*	606	[46]
IDL31F	CTGTGCTACACCTAGAGATAGGTGG	*L. delbrueckii*	184	[46]
IDL42R	ATTTCAAGTTGAGTCTCTCTCTC	*L. gasseri*	272	[46]
IDL52F	ACCTGATTGACGATGGATCACCAGT	*L. reuteri*	1105	[46]
DL62R	CTAGTGGTAACAGTTGATTAAAACTGC	*L. plantarum*	428	[46]
IDL73R	GCCAACAAGCTATGTGTTCGCTTGC	*L. rhamnosus*	448	[46]
Enterococcus				
Ent1	TACTGACAAACCATTCATGATG	Genus-specificity	112	[47]
Ent2	AACTTCGTCACCAACGCGAAC			
FL1	ACTTATGTGACTAACTTAACC	*E. faecalis*	360	[48]
FL2	TAATGGTGAAATCTTGGTTTGG			
FM1	GAAAAAACAATAGAAGAATTAT	*E. faecium*	215	[48]
FM2	TGCTTTTTTGAATTCTTCTTTA			
GA1	TTACTTGCTGATTTTGATTCG	*E. gallinarum*	173	[48]
GA2	TGAATTCTTCTTTGAAATCAG			
CA1	TCCTGAATTAGGTGAAAAAAC	*E. casseliflavus*	288	[48]
CA2	GCTAGTTTACCGTCTTTAACG			
HI1	CTTTCTGATATGGATGCTGTC	*E. hirae*	187	[48]
HI2	TAAATTCTTCCTTAAATGTTG			
DU1	CCTACTGATATTAAGACAGCG	*E. durans*	295	[48]
DU2	TAATCCTAAGATAGGTGTTTG			
Clostridium				

16SA	GAGAGTTTGATCCTGGCTCAG	Genus-specificity	800	[49]
16SB	GTGGACTACCAGGGTATCTAATCC			
ClPER-F	AGATGGCATCATCATTCAAC	*C. perfringens*	793	[49]
ClPER-R	GCAAGGGATGTCAAGTGT			
ClBIF-F	CAAGTCGAGCGATCTCT	*C. bifermentans*	564	[49]
ClBIF-R	CCTGCACTCAAGTTCTCT			
ClDIF-F	CTTGAATATCAAAGGTGAGCCA	*C. difficile*	1085	[49]
ClDIF-R	CTACAATCCGAACTGAGAGTA			
ClSOR-F	TCGAGCGACCTTCGG	*C. sordellii*	944	[49]
ClSOR-R	CACCACCTGTCACCAT			
ClCLO-F	GAAGTTTTCGGATGGAATCTTGA	*C. clostridiiforme*	762	[49]
ClCLO-R	CACCGAAGGCTTTGCC			
ClNEX-F	ATGGCACAGTGTAAAAACTCCG	*C. nexile*	1054	[48]
ClNEX-R	TTGCTTCCCCTCACAGGT			
ClSPH-F	GAAGTTTTCGGACGGATTTTGA	*C. sphenoides*	1058	[49]
ClSPH-R	AGAGTGCCCAACTTGACC			
ClIND-F	GACTGCTTTGGAAACTGTGT	*C. indolis*	369	[49]
ClIND-R	AGGCCCCGTTACGGA			
ClINN-F	GGGGGATAATTATGGATCAC	*C. innocuum*	241	[49]
ClINN-R	GTCGCTGCTCTTTGTGG			
ClRAM-F	GTGACCGTATTAAAAGTGCCT	*C. ramosum*	298	[49]
ClRAM-R	TACCGTCACTCGGCTAC			
ClCOC-F	GTAATACATAAGTAACCTGGCCTTT	*C. cocleatum*	373	[49]
ClCOC-R	CTCGGATGTCATTTCCTCC			

Table S2. Primers used for detection of AMR genes (*n* = 112).

Gene	Primer	Primer Sequences (5′–3′)	Annealing Temperature (°C)	Product Size (bp)	Reference
Beta-lactams					
*bla*PSE-1	blaPSE1-F	GCAAGTAGGGCAGGCAATCA	55	422	[50]
	blaPSE1-R	GAGCTAGATAGATGCTCACAA			
*bla*TEM	blaTEM-F	ATCAGTTGGGTGCACGAGTG	55	608	[50]
	blaTEM-R	ATCAGTTGGGTGCACGAGTG			
*bla*SHV	blaSHV-F	TTCGCCTGTGTATTATCTCCCTG	50	854	[26]
	blaSHV-R	TTAGCGTTGCCAGTGYTG			
*bla*CMY-1	blaCMY-1F	GTGGTGGATGCCAGCATCC	58	915	[26]
	blaCMY-1R	GGTCGAGCCGGTCTTGTTGAA			
*bla*CMY-2	blaCMY-2F	GCACTTAGCCACCTATACGGCAG	58	758	[26]
	blaCMY-2F	GCTTTTCAAGAATGCGCCAGG			
*bla*CTX-M universal	blaCTX-MF	CGATGTGCAGTACCAGTAA	60	585	[19]
	blaCTX-MR	AGTGACCAGAATCAGCGG			
*bla*CTX-M group 1	blaCTX-M group1-IF	TTAGGAARTGTGCCGCTGYA	60	688	[51]
	blaCTX-M group1-IR	CGATATCGTTGGTGGTRCCAT			
*bla*CTX-M group 2	blaCTX-M group2-IF	CGTTAACGGCACGATGAC	60	404	[51]
	blaCTX-M group2-IR	CGATATCGTTGGTGGTRCCAT			
*bla*CTX-M group 9	blaCTX-M group9-IF	TCAAGCCTGCCGATCTGGT	60	561	[51]
	blaCTX-M group9-IR	TGATTCTCGCCGCTGAAG			
*bla*CTX-M group 8/25	blaCTX-M group8-IF	AACRCRCAGACGCTCTAC	60	326	[51]
	blaCTX-M group8-IR	TCGAGCCGGAASGTGTYAT			
*bla*CTX-M-15	blaCTX-M 15-IF	CACACGTGGAATTTAGGGACT	56	995	[52]
	blaCTX-M 15-IR	GCCGTCTAAGGCGATAAACA			
*bla*VEB	MultiVEB_for	CATTTCCCGATGCAAAGCGT	60	648	[51]

	MultiVEB_rev	CGAAGTTTCTTTGGACTCTG			
bla$_{GES}$	MultiGES_for	AGTCGGCTAGACCGGAAAG	60	399	[51]
	MultiGES_rev	TTTGTCCGTGCTCAGGAT			
bla$_{PER}$	MultiPER_for	GCTCCGATAATGAAAGCGT	60	520	[51]
	MultiPER_rev	TTCGGCTTGACTCGGCTGA			
bla$_{ACC}$	MultiCaseACC_for	CACCTCCAGCGACTTGTTAC	60	346	[51]
	MultiCaseACC_rev	GTTAGCCAGCATCACGATCC			
bla$_{FOX}$	MultiCaseFOX_for	CTACAGTGCGGGTGGTTT	60	126	[51]
	MultiCaseFOX_rev	CTATTTGCGGCCAGGTGA			
bla$_{MOX}$	MultiCaseMOX_for	GCAACAACGACAATCCATCCT	60	895	[51]
	MultiCaseMOX_rev	GGGATAGGCGTAACTCTCCCAA			
bla$_{DHA}$	MultiCaseDHA_for	TGATGGCACAGCAGGATATTC	60	997	[51]
	MultiCaseDHA_rev	GCTTTGACTCTTTCGGTATTCG			
bla$_{CIT}$	MultiCaseCIT_for	CGAAGAGGCAATGACCAGAC	60	538	[51]
	MultiCaseCIT_rev	ACGGACAGGGTTAGGATAGY			
bla$_{EBC}$	MultiCaseEBC_for	GGCACCAGATTCAACTTTCAAG	60	683	[51]
	MultiCaseEBC_rev	GACCCCAAGTTTCCTGTAAGTG			
bla$_{OXA\text{-}1\text{-}like}$	MultiTSO-O_for	GGCACCAGATTCAACTTTCAAG	60	564	[51]
	MultiTSO-O_rev	GACCCCAAGTTTCCTGTAAGTG			
blaZ	stau-*blaZ*-F	CAAAGATGATATAGTTGCTTATTCTCC	50	421	[52]
	stau-*blaZ*-R	TGCTTGACCACTTTTATCAGC			
mecA	mecA-F1	TGGTATGTGGAAGTTAGATTGGGAT	60	155	[53]
	mecA-R1	CTAATCTCATATGTGTTCCTGTATTGGC			
bla$_{KPC}$	KPC-Fm	CGTCTAGTTCTGCTGTCTTG	52	798	[54]
	KPC-Rm	CTTGTCATCCTTGTTAGGCG			
bla$_{NDM}$	NDM-F	GGTTTGGCGATCTGGTTTTC	52	621	[54]
	NDM-R	CGGAATGGCTCATCACGATC			
bla$_{OXA\text{-}48}$	OXA-F	GCGTGGTTAAGGATGAACAC	52	438	[54]
	OXA-R	CATCAAGTTCAACCCAACCG			
bla$_{IMP}$	IMP-F	GGAATAGAGTGGCTTAAYTCTC	52	232	[54]
	IMP-R	GGTTTAAYAAAACAACCACC			
bla$_{VIM}$	VIM-F	GATGGTGTTTGGTCGCATA	52	390	[54]
	VIM-R	CGAATGCGCAGCACCAG			
Quinolones					
qnrA	qnrA-F	ATTTCTCACGCCAGGATTTG	53	516	[55]
	qnrA-R	GATCGGCAAAGGTTAGGTCA			
qnrB	qnrB-F	GATCGTGAAAGCCAGAAAGG	53	469	[55]
	qnrB-R	ACGATGCCTGGTAGTTGTCC			
qnrS	qnrS-F	ACGACATTCGTCAACTGCAA	53	417	[55]
	qnrS-R	TAAATTGGCACCCTGTAGGC			
qepA	QepA-F	GCAGGTCCAGCAGCGGGTAG	60	199	[56]
	QepA-R	CTTCCTGCCCGAGTATCGTG			
aac(6′)-Ib-cr	AAC(6′)-Ib-F	TTGCGATGCTCTATGAGTGGCTA	55	482	[22]
	AAC(6′)-Ib-R	CTCGAATGCCTGGCGTGTTT			
qnrC	qnrC-F	GGGTTGTACATTTATTGAATC	50	447	[57]
	qnrC-R	TCCACTTTACGAGGTTCT			
qnrD	qnrD fw	CGAGATCAATTTACGGGGAATA	50	582	[21]
	qnrD rev	AACAAGCTGAAGCGCCTG			
oqxA	oqxAF	CTCGGCGCGATGATGCT	55	392	[58]
	oqxAR	CCACTCTTCACGGGAGACGA			
oqxB	oqxBs	TTCTCCCCCGGCGGGAAGTAC	55	512	[58]
	oqxBa2	CTCGGCCATTTTGGCGCGTA			
Aminoglycosides					
aadA1	aadA1-F	CTCCGCAGTGGATGGCGG	55	631	[50]
	aadA1-R	GATCTGCGCGCGAGGCCA			
aadA2	aadA2-F	CATTGAGCGCCATCTGGAAT	55	500	[50]
	aadA2-R	ACATTTCGCTCATCGCCGGC			
aadB	aadB-F	CTAGCTGCGGCAGATGAGC	57	300	[50]

	aadB-R	CTCAGCCGCCTCTGGGCA			
aad(E)	aadEI	GCAGAACAGGATGAACGTATTCG	55	369	[59]
	aadEII	ATCAGTCGGAACTATGTCCC			
aac(6′)-aph(2′′)	aac(6')aph(2'')F	CCAAGAGCAATAAGGGCATA	60	222	[20]
	aac(6')aph(2'')R	CACTATCATAACCACTACCG			
strA	strA-F	TGGCAGGAGGAACAGGAGG	57	405	[14]
	strA-R	AGGTCGATCAGACCCGTGC			
strB	strB-F	GCGGACACCTTTTCCAGCCT	57	621	[60]
	strB-R	TCCGCCATCTGTGCAATGCG			
armA	armA-F	CCGAAATGACAGTTCCTATC	55	846	[61]
	armA-R	GAAAATGAGTGCCTTGGAGG			
rmtB	rmtB-F	ATGAACATCAACGATGCCCT	55	769	[62]
	rmtB-R	CCTTCTGATTGGCTTATCCA			
aac(3)-I	aac(3)-I F	GGGCATCATTCGCACATGTAGGC	64	429	[63]
	aac(3)-I R	CATCACTTCTTCCCGTATGCCC			
aac(3)-II	aac(3)-II F	TGAAACGCTGACGGAGCCTC	58	369	[64]
	aac(3)-II R	GTCGAACAGGTAGCACTGAG			
aac(3)-III	aac(3)-III F	GTGCATCGCAGCGCAAACCCC	64	436	[63]
	aac(3)-III R	CAAGCCACTGCACCGCAAACCG			
aac(3)-IV	aac(3)-IV F	GTGTGCTGCTGGTCCACAGC	58	628	[64]
	aac(3)-IV R	AGTTGACCCAGGGCTGTCGC			
aph(2′′)-Ib	aph2-Ib-F	CTTGGACGCTGAGATATATGAGCAC	55	867	[65]
	aph2-Ib-R	GTTTGTAGCAATTCAGAAACACCCTT			
aph(2′′)-Ic	aph2-Ic-F	CCACAATGATAATGACTCAGTTCCC	55	444	[65]
	aph2-Ic-R	CCACAGCTTCCGATAGCAAGAG			
aph(2′′)-Id	aph2-Id-F	GTGGTTTTTACAGGAATGCCATC	55	641	[65]
	aph2-Id-R	CCCTCTTCATACCAATCCATATAACC			
aph(3′)-IIIa	aph3-IIIa-F	GGCTAAAATGAGAATATCACCGG	55	523	[65]
	aph3-IIIa-R	CTTTAAAAAATCATACAGCTCGCG			
ant(4′)-Ia (aadD)	ant4-Ia-F	CAAACTGCTAAATCGGTAGAAGCC	55	294	[65]
	ant4-Ia-R	GGAAAGTTGACCAGACATTACGAACT			
Chloramphenicol					
catA	catA-F	CCAGACCGTTCAGCTGGATA	55	454	[50]
	catA-R	CATCAGCACCTTGTCGCCT			
catB	catB-F	CGGATTCAGCCTGACCACC	55	461	[50]
	catB-R	ATACGCGGTCACCTTCCTG			
cmlA	cmlA-F	TGGACCGCTATCGGACCG	57	641	[66]
	cmlA-R	CGCAAGACACTTGGGCTGC			
florR	florR-F	ATGGTGATGCTCGGCGTGGGCCA	58	800	[67]
	florR-R	GCGCCGTTGGCGGTAACAGACACCGTGA			
Macrolides					
ermA	ermAI	TCTAAAAAGCATGTAAAAGAA	52	645	[68]
	ermAII	CTTCGATAGTTTATTAATATTAGT			
ermB	ermBI	GAAAAGGTACTCAACCAAATA	52	638	[68]
	ermBII	AGTAACGGTACTTAAATTGTTTAC			
ermC	ermCI	TCAAAACATAATATAGATAAA	52	643	[68]
	ermCII	GCTAATATTGTTTAAATCGTCAAT			
mefA	mef(A)-FW	CAATATGGGCAGGGCAAG	62	317	[28]
	mef(A)-RW	AAGCTGTTCCAATGCTACGG			
mph(A)	mphAF	GTGAGGAGGAGCTTCGCGAG	60	403	[69]
	mphAR	TGCCGCAGGACTCGGAGGTC			
mph(B)	mphBF	GATATTAAACAAGTAATCAGAATAG	58	494	[69]
	mphBR	GCTCTTACTGCATCCATACG			
mph(C)	mphCF	ATGACTCGACATAATGAAAT	45	900	[70]
	mphCR	CTACTCTTTCATACCTAACTC			
ere(A)	ereAF	GCCGGTGCTCATGAACTTGAG	60	420	[69]
	ereAR	CGACTCTATTCGATCAGAGGC			
ere(B)	ereBF	TTGGAGATACCCAGATTGTAG	55	537	[69]

	ereBR	GAGCCATAGCTTCAACGC			
ermF	F1	CGGGTCAGCACTTTACTATTG	50	466	[71]
	F2	GGACCTACCTCATAGACAAG			
msrA	msrA F	GGCACAATAAGAGTGTTTAAAGG	60	940	[72]
	msrA R	AAGTTATATCATGAATAGATTGTCCTGTT			
msrB	msrB F	TATGATATCCATAATAATTATCCAATC	60	595	[72]
	msrB R	AAGTTATATCATGAATAGATTGTCCTGTT			
ermTR	TR3	CAATAAACAAGATAAAATAATAG	47	531	[73]
	TR4	CTTTTTGTAGTCCTTCTTTAA			
Trimethoprim					
dfrA1	dfrA1-F	CAATGGCTGTTGGTTGGAC	55	254	[50]
	dfrA1-R	CCGGCTCGATGTCTATTGT			
dfrA10	dfrA10-F	TCAAGGCAAATTACCTTGGC	57	432	[61]
	dfrA10-R	ATCTATTGGATCACCTACCC			
dfrA12	dfrA12-F	TTCGCAGACTCACTGAGGG	55	330	[50]
	dfrA12-R	CGGTTGAGACAAGCTCGAAT			
dfrA5	dfr5-f	AGCTACTCTTTAAAGCCTTGACGTA	55	341	[74]
	dfr5-r	GTGTTGCTCAAAAACAACTTCG			
dfrA7	dfr7&17-f	ACATTTGACTCTATGGGTGTTCTTC	55	227	[74]
	dfr7-r	ACCTCAACGTGAACAGTAGACAAAT			
dfrA17	dfr7&17-f	ACATTTGACTCTATGGGTGTTCTTC	55	171	[74]
	dfr17-r	TCTCTGGCGGGGGTCAAATCTAT			
dfrA14	dfrA14-F	TTAACCCAGGATGAGAACCT	52	510	[75]
	dfrA14-R	CGATTGCATAGCTTTGTTAA			
dfr18	dfr18-F	TGGGTAAGACACTCGTCATGGG	43	389	[76]
	dfr18-R	ACTGCCGTTTTCGATAATGTGG			
dfrA8	dfrA8-F	GAGCTTCCGGGTGTTCGTGAC	55	247	[77]
	dfrA8-R	CTTCCATGCCATTCTGCTCGTAGT			
Sulfonamides					
sul1	Sul1-F	CGGACGCGAGGCCTGTATC	57	591	[66]
	Sul1-R	GGGTGCGGACGTAGTCAGC			
sul2	sul2-F	GCGCAGGCGCGTAAGCTGAT	57	514	[61]
	sul2-R	CGAAGCGCAGCCGCAATTC			
sul3	sul3-F	GGGAGCCGCTTCCAGTAAT	57	500	[66]
	sul3-R	TCCGTGACACTGCAATCATTA			
Tetracyclines					
tetA	tetA-F	GCTGTCGGATCGTTTCGG	55	658	[50]
	tetA-R	CATTCCGAGCATGAGTGCC			
tetB	tetB-F	CTGTCGCGGCATCGGTCAT	55	615	[50]
	tetB-R	CAGGTAAAGCGATCCCACC			
tetK	tetKI	TTGAGCTGTCTTGGTTCA	50	352	[14]
	tetKII	CAATACCTACGATATCTA			
tetL	tet(L)I	TGGTCCTATCTTCTACTCATTC	53	385	[78]
	tet(L)II	TTCCGATTTCGGCAGTAC			
tetM	tet(M)I	GGTGAACATCATAGACACGC	55	401	[78]
	tet(M)II	CTTGTTCGAGTTCCAATGC			
tetO	tet(O)I	AGCGTCAAAGGGGAATCACTATCC	55	1723	[14]
	tet(O)II	CGGCGGGGTTGGCAAATA			
tetS	tet(S)I	ATCAAGATATTAAGGAC	55	573	[79]
	tet(S)II	TTCTCTATGTGGTAATC			
tetW	TetW-FW	GAGAGCCTGCTATATGCCAGC	52	168	[80]
	TetW-RW	GGGCGTATCCACAATGTTGAC			
tet(C)	tetC-F	CTTGAGAGCCTTCAACCCAG	55	418	[81]
	tetC-R	ATGGTCGTCATCTACCTGCC			
tet(D)	tetD-F	AAACCATTACGGCATTCTGC	55	787	[81]
	tetD-R	GACCGGATACACCATCCATC			
tet(E)	tetE-F	AAACCACATCCTCCATACGC	55	278	[81]
	tetE-R	AAATAGGCCACAACCGTCAG			

tet(G)	tetG-F	GCTCGGTGGTATCTCTGCTC	55	468	[81]
	tetG-R	AGCAACAGAATCGGGAACAC			
tet(Q)	tetQ-F	TTATACTTCCTCCGGCATCG	55	904	[81]
	tetQ-R	ATCGGTTCGAGAATGTCCAC			
tet(X)	tetX-F	CAATAATTGGTGGTGGACCC	55	468	[81]
	tetX-R	TTCTTACCTTGGACATCCCG			
tet(30)	tet(30)-F	CCGTCATGCAATTTGTGTTC	60	550	[82]
	tet(30)-R	TAGAGCACCCAGATCGTTCC			
tet(32)	tet(32)-F	GAACCAGATGCTGCTCTT	57	620	[83]
	tet(32)-R	CATAGCCACGCCCACATGAT			
tet(O/W/32/O)	tetWF	GGAGGAAAATACCGACATA	50	729	[84]
	tet32R	CTCTTTCATAGCCACGCC			
Polymyxins					
mcr-1	MCR1-IF	AGTCCGTTTGTTCTTGTGGC	58	320	[85]
	MCR1-IR	AGATCCTTGGTCTCGGCTTG			
mcr-2	MCR2-IF	CAAGTGTGTTGGTCGCAGTT	58	715	[85]
	MCR2-IR	TCTAGCCCGACAAGCATACC			
mcr-3	MCR3-IF	AAATAAAAATTGTTCCGCTTATG	58	929	[85]
	MCR3-IR	AATGGAGATCCCCGTTTTT			
mcr-4	MCR4-IF	TCACTTTCATCACTGCGTTG	58	1116	[85]
	MCR4-IR	TTGGTCCATGACTACCAATG			
mcr-5	MCR5-IF	ATGCGGTTGTCTGCATTTATC	58	1644	[85]
	MCR5-IR	TCATTGTGGTTGTCCTTTTCTG			
mcr-6	MCR-6F	GTCCGGTCAATCCCTATCTGT	55	556	[86]
	MCR-6R	ATCACGGGATTGACATAGCTAC			
mcr-7	MCR-7F	TGCTCAAGCCCTTCTTTTCGT	55	894	[86]
	MCR-7R	TTCATCTGCGCCACCTCGT			
mcr-8	MCR-8F	AACCGCCAGAGCACAGAATT	60	667	[86]
	MCR-8R	TTCCCCCAGCGATTCTCCAT			
Vancomycin					
vanA	vanA1	GGGAAAACGACAATTGC	54	732	[87]
	vanA2	GTACAATGCGGCCGTTA			
vanB	vanB1	ATGGGAAGCCGATAGTC	54	635	[87]
	vanB2	GATTTCGTTCCTCGACC			
vanC	vanC1	GGTATCAAGGAAACCTC	54	822	[87]
